# Update on the outcome of M‐protein screening program of multiple myeloma in China: A 7‐year cohort study

**DOI:** 10.1002/cam4.6859

**Published:** 2023-12-22

**Authors:** Wenjing Wang, Jing Li, Yang Yang, Feifei Chen, Tianhong Xu, Pu Wang, Yawen Wang, Aziguli Maihemaiti, Liang Ren, Tianwei Lan, Panpan Li, Chi Zhou, Peng Liu

**Affiliations:** ^1^ Department of Hematology Zhongshan Hospital Fudan University Shanghai China

**Keywords:** cancer early detection, frontline therapy, M‐protein screening, multiple myeloma, patient outcomes

## Abstract

**Background:**

To improve the early detection rate of multiple myeloma (MM), the M‐protein screening system has been performed in the hospital population at Zhongshan Hospital Fudan University since 2014, with electrophoretic‐based monoclonal immunoglobulin (M‐protein) screening integrated into the blood biochemistry panel. This study updated 7‐year follow‐up findings of MM patients diagnosed by screening‐driven and symptom‐driven approaches.

**Method**s**:**

The retrospective study compared the characteristics and outcomes of patients diagnosed through two patterns by reviewing the plasma cell disease database from January 2014 to October 2021. The screening‐driven group included patients diagnosed through the screening system during workups of unrelated medical conditions or routine checkups. In contrast, patients who visited or were referred to the hematological department due to myeloma‐related end‐organ damage were categorized into the symptom‐driven group.

**Results:**

There were 3,110,218 serum protein electrophoresis (SPEP) tests performed during 7 years, with 1.95% (60,609) patients yielding positive SPEP results. Of 911 confirmed MM cases (excluding concurrent amyloidosis), 366 were assigned to the screening‐driven group, while 545 were to the symptom‐driven group. Compared to the symptom‐driven group, the screening group had more IgG subtypes, earlier International Stage System stages, fewer disease‐related symptoms, lower ECOG scores, less extramedullary disease, a lower percentage of bone marrow plasma cells, and a lower level of lactate dehydrogenase. Frontline response results of two groups were similar. Patients detected through screening had a significantly improved median progression‐free survival (PFS) than the symptom‐driven group (62.2 vs. 24.9 months, *p* < 0.001, HR: 2.12, 95% CIs: 1.69–2.65), with median follow‐ups of 32.6 and 27.4 months. Furthermore, the median overall survival (OS) was significantly longer in patients of the screening group (not reached vs. 62.3 months, *p <* 0.001, HR: 2.49, 95% CIs: 1.81–3.41). After being adjusted for well‐acknowledged myeloma prognostic factors, the screening‐driven diagnostic pattern remained an independent prognostic factor indicating improved PFS and OS in MM patients.

**Conclusion:**

Routine M‐protein screening for MM in the hospital population results in an earlier diagnosis and better patient outcomes.

## Graphical abstract

This study updated 7‐year follow‐up findings of MM patients diagnosed by screening‐driven and symptom‐ driven approaches in the hospital population. The result confirmed the efficacy of the screening system at Zhongshan Hospital Fudan University, thereby demonstrating the viability of this screening program in China.

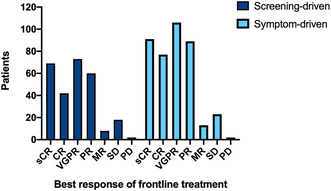

## 1 INTRODUCTION

Multiple myeloma (MM) is the second most common hematological malignancy, accounting for 1%–1.8% of all cancers.^1^ The principal clinical signs are anemia, renal insufficiency, hypercalcemia, and bone damage, which are caused by the clonal proliferation of plasma cells in the bone marrow.^2^ Currently, the reported incidence of myeloma in Asia is comparatively lower than that in Western countries.^3^ Besides racial and ethnic factors, economic and cultural differences may also contribute to the disparity.^4^ Meanwhile, the current situation of MM in China has various disparities between provinces. For example, Hong Kong had a higher incidence rate than Gansu.^5^ The under‐recognition and uneven medical input on illness screening in many regions of the country may be one of significant reasons.^6^, ^7^

The monoclonal immunoglobulin, or M‐protein, which is produced excessively, is a biomarker for monitoring the disease status and treatment response of MM.^8^, ^9^ The iStopMM (Iceland Screens, Treats or Prevents Multiple Myeloma) study, which involved Icelanders over the age of 40, discovered a high prevalence of smoldering MM (SMM) by screening the general population for M‐protein.^10^, ^11^ Additionally, several studies have demonstrated that clinical follow‐ups of monoclonal gammopathy of undetermined significance (MGUS) detected through screening leads to better outcomes in MM, with serum protein electrophoresis (SPEP) being a standard screening method.^12^, ^13^ However, there still exists a discrepancy between the limited evidence of the benefits of MM screening and the high cost of screening a national population.^14^ Screening a specific population appears to be a more practical approach, relatively. For example, studies conducted in the USA and the UK have showed the advantages of screening individuals at high risk for MM.^15^, ^16^

As the first institution to perform M‐protein screening in China, Zhongshan Hospital Fudan University, a national medical center, combined electrophoretic‐based tests with blood liver function tests. Since 2014, our hospital has implemented a screening program aimed at improving the detection rate of MM. In our previous study,^17^ we demonstrated that M‐protein screening improved the early diagnosis rate of MM and prolonged their overall survival (OS) in the hospital population. In this study, we evaluated the predictive role of a diagnostic pattern in newly diagnosed MM (NDMM) patients and assessed the utility of electrophoretic‐based screening in the hospital population by analyzing its impact on patient outcomes over a 7‐year follow‐up period.

## 2 MATERIALS AND METHODS

**FIGURE 1:**
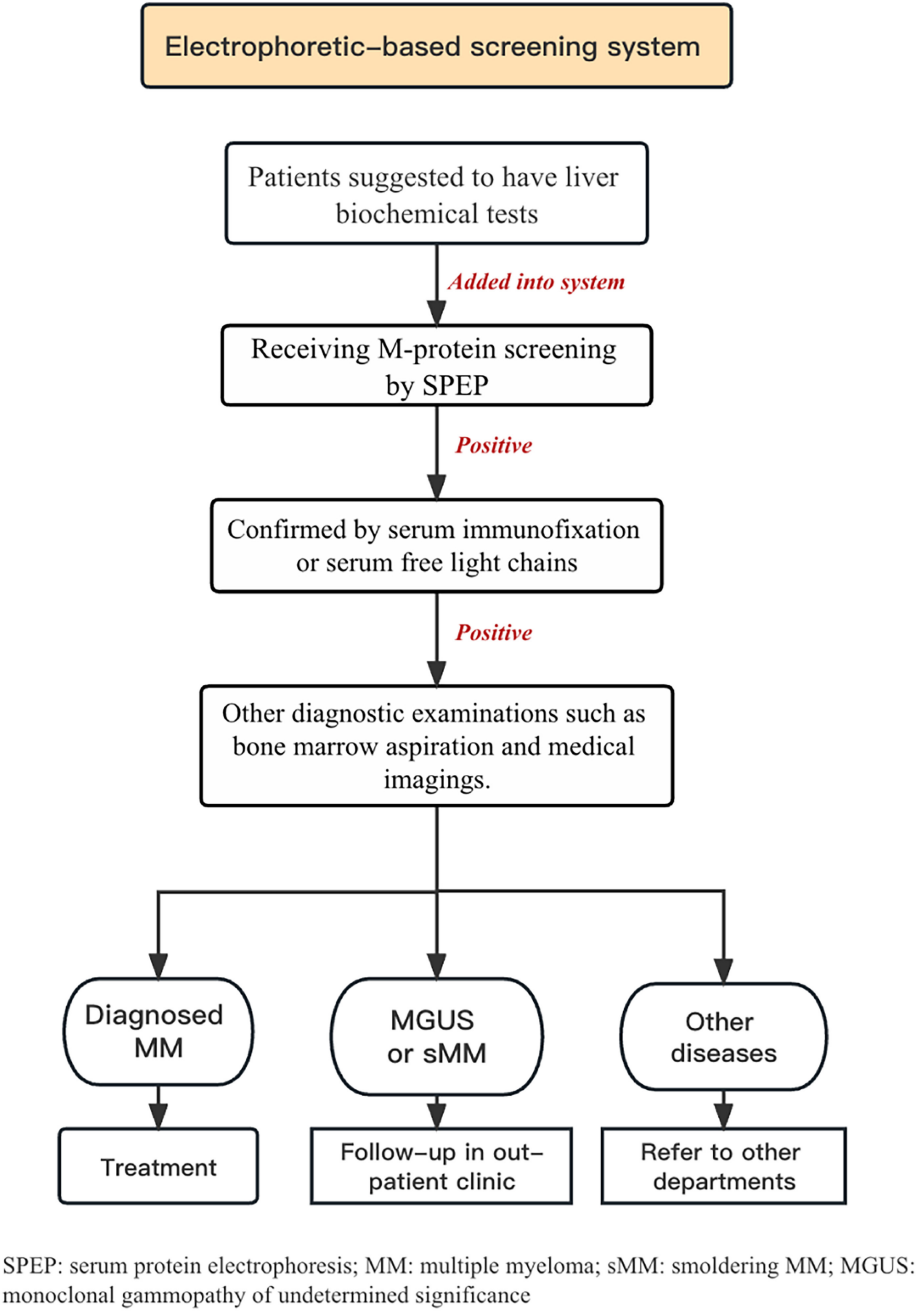
Paradigm of electrophoretic‐based M‐protein screening system for multiple myeloma.

**FIGURE 2:**
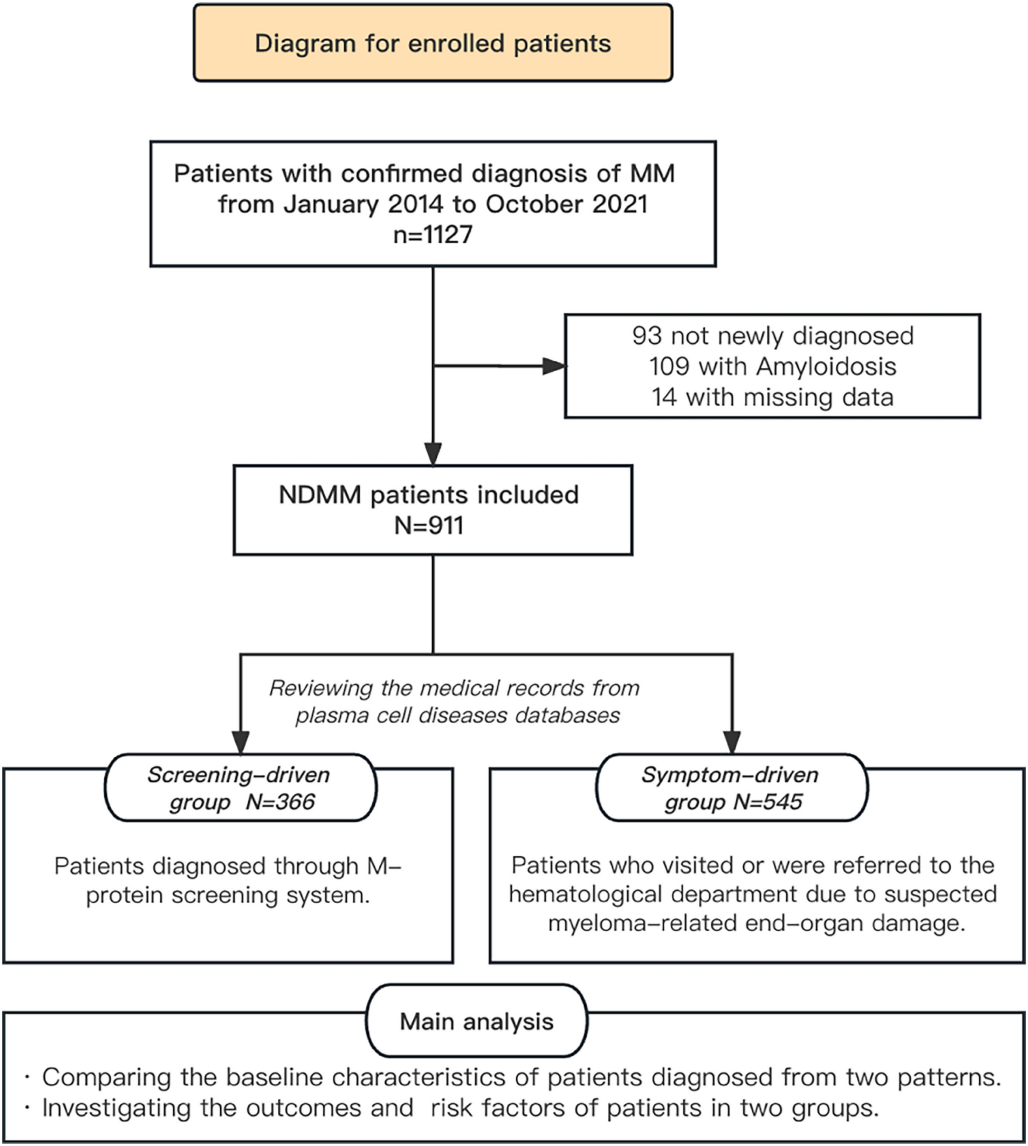
Study flowchart for cohort selection. The study enrolled patients aging over 18 from January 2014 to October 2021 with confirmed diagnose of multiple myeloma (MM) at Zhongshan Hospital Fudan University. By reviewing medical information in database, 93 of patients who were not newly diagnosed at our center, 109 patients concurrent with diagnosis of amyloidosis (AL) and 14 patients without baseline information were excluded. A total of 911 cases were included and divided into two groups. NDMM, newly diagnosed MM.

This is a retrospective study of patients with NDMM from January 2014 to October 2021 at Zhongshan Hospital Fudan University. We obtained medical records of patients by the Investigator Initiated Trial‐Electronic Data Capture system (Zhejiang Taimei Medical Technology Co., Ltd). The terms “M protein”, “multiple myeloma”, and “plasmacytoma” were identified as keywords to capture relevant cases. The diagnosis of active MM was confirmed by International Myeloma Working Group (IMWG) updated criteria.^18^ Precursor MM is defined as a medical history of MGUS or SMM diagnosed at least 3 months prior to the diagnosis of active MM according to the updated IMWG criteria. Patients concurrent with a diagnosis of amyloidosis (AL) were excluded because the malignant cells involved and the manner in which the disease progresses in MM differ from those in AL.^19^ Then all confirmed cases were divided into two groups according to their diagnostic pattern. The screening‐driven group included patients who were diagnosed through M‐protein screening system. This screening system has been conducted in our center since 2014 (Figure 1). Since SPEP was routinely incorporated into the test panel, patients who were going to have a liver function biochemistry test would be added to the screening process. Capillary electrophoresis is used for samples by Sebia Capillarys 3 TERA (Sebia, France). The minimum detection limit of liquid‐based capillary electrophoresis technology is 0.18 g/L M‐protein. A prompt of M‐protein detection would pop up in the biochemistry test report if a patient had a positive SPEP result, and he/she would be suggested to receive immunofixation electrophoresis test, thereby entering the follow‐up process. Before the initiation of M‐protein screening, patients in our hospital were usually diagnosed when severe complications occurred. The symptom‐driven group here included individuals who visited or were referred to the hematological department due to suspected myeloma‐related end‐organ damage (Figure 2).

Then the study analyzed the characteristics and outcomes of patients in two groups by reviewing data from medical records. Baseline information contained age, gender, M‐protein subtype, International Stage System (ISS), Eastern Cooperative Oncology Group (ECOG) scores, extramedullary disease (EMD), tumor burden (e.g., lactate dehydrogenase (LDH), bone marrow plasma cells (BMPC)), complications (e.g., hypercalcemia, renal insufficiency, anemia and bone lesion), chromosomal abnormalities, and cytogenetic abnormalities (CAs). High‐risk (HR) CAs here included Del 17p, Amp/Gain 1q21, t(4;14), and t(14;16). The response to therapy and progression were assessed according to the IMWG criteria.^20^ Median OS and progression‐free survival (PFS) were used to describe the survival outcomes of MM patients. PFS was defined as the duration time between diagnosis and progression of the disease or death from any cause. OS was defined as the time from the start of diagnosis to last contact or death due to any cause. Using univariable and multivariable cox proportional hazards models, the prognostic significance of risk factors was estimated.

The study was conducted following the Declaration of Helsinki. All participants provided written informed consent for using anonymized medical data in research. The clinical ethics committee of Zhongshan Hospital Fudan University approved the study procedure (B2017‐031R, B2023‐185R).

## 3 STATISTICAL ANALYSIS

Means with standard deviations or medians with ranges were calculated for continuous variables, whereas frequencies with percentages were reported for categorical variables. Comparison of variables was conducted by Student's *t*‐test, Mann–Whitney *U*‐test or chi‐squared test. We constructed Kaplan–Meier curve for survival analyses. Median follow‐up time was calculated by reversed Kaplan–Meier method.^21^ The hazard ratio (HR) with 95% confidence intervals (CIs) was computed using cox proportional hazards models. All statistical analyses were conducted by SPSS software (version 25.0) and R packages in R/Bioconductor (version 3.6.1). *p* < 0.05 was considered statistically significant.

## 4 RESULTS

### 4.1 General information

**FIGURE 3:**
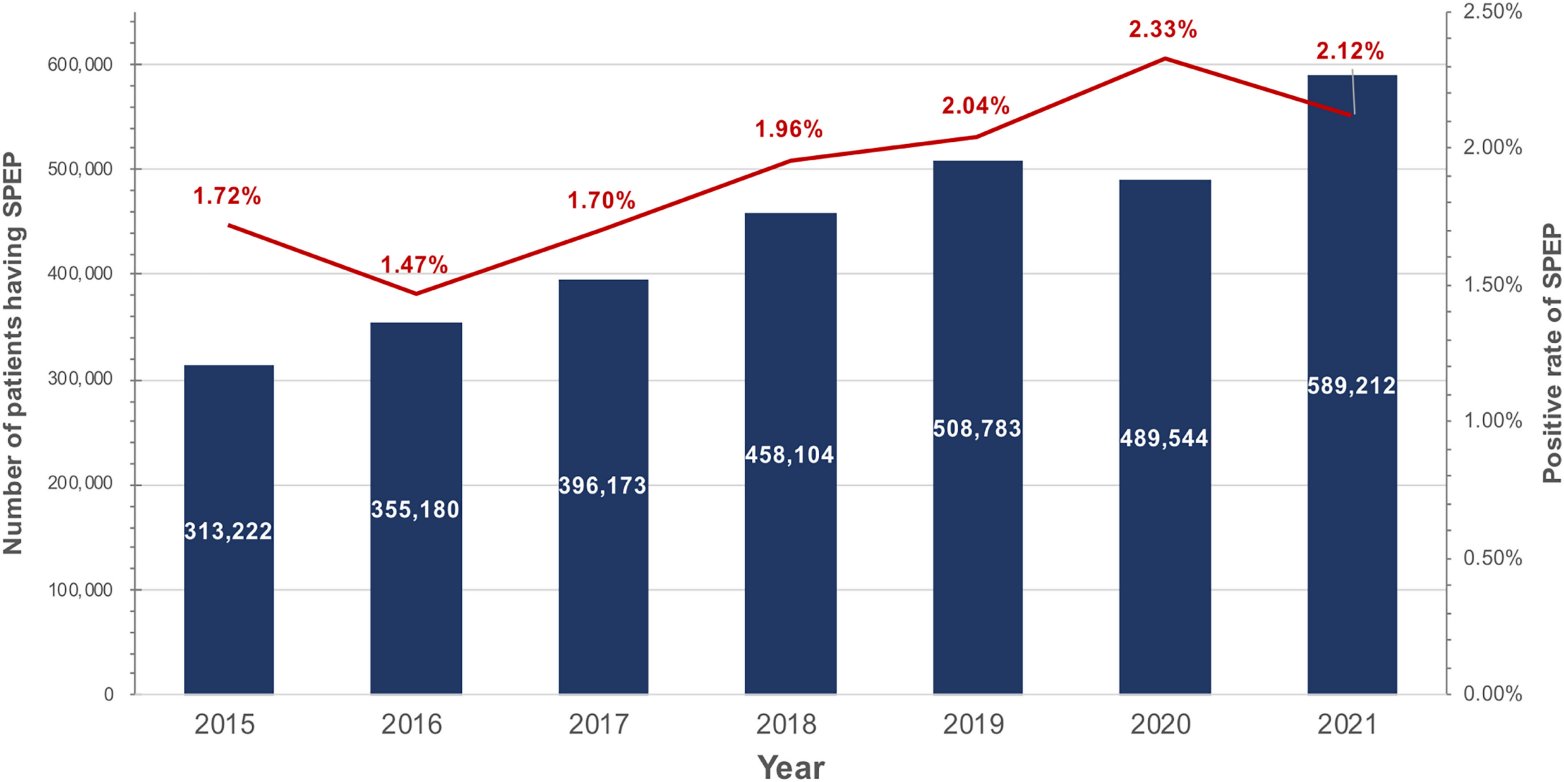
The overview of serum protein electrophoresis (SPEP) results at Zhongshan Hospital, Fudan University. A total of 3,110,218 SPEP tests were conducted between 2015 and 2021, among which 1.95% (60,609) of patients yielded positive SPEP results. The annual positivity rate ranges from 1.47% to 2.33%.

Due to the rising number of patient visits to our hospital over the previous 7‐year, the M‐protein screening system was gradually scaled up. There were 3,110,218 SPEP tests performed during the past 7 years, with 1.95% (60,609) patients yielding positive SPEP results. The annual positivity rate ranges from 1.47% to 2.33% (Figure 3). From January 2014 to October 2021, 1127 patients with a confirmed diagnosis of MM at Zhongshan Hospital Fudan University were finally collected. According to our data, the annual incidence of NDMM was 31.9 per 100,000 hospital screening patients. By reviewing medical information in the database, 93 patients who were not newly diagnosed at our center, 109 patients concurrent with diagnosis of AL and 14 patients without baseline information were excluded (Figures 1 and 2). A total of 911 NDMM cases were included and divided into two groups. Compared to the 151 versus 184 reported in 2019,^17^ there were 366 screening‐driven patients and 545 symptom‐driven patients.

### 4.2 Patient characteristics

Clinical characteristics of patients diagnosed in two cohorts are summarized in Table 1. The median age was 66 years (range, 35–87 years) in the screening‐driven group and 65 (range, 32–87 years) in the symptom‐driven group, with 188 (51.4%) patients above the age of 65 in the screening‐driven one and 253 (46.4%) in the other (*p > 0.05*). The sex distribution in both groups was similar. There were significant differences between the two groups in terms of M‐protein subtype, disease stage, ECOG score, EMD, tumor burden, complications and HRCAs. The screening group had more patients with IgG phenotype (*p = 0.001*), early ISS stages (*p < 0.001*), low ECOG scores (*p < 0.001*), non‐EMD (*p < 0.001*), low percentage of BMPC (*p < 0.001*), and normal level of LDH (*p = 0.018*). Fewer disease‐related “CRAB” complications (hypercalcemia, renal insufficiency, anemia, and bone lesion) were found in patients of the screening group compared to the symptom‐driven group. Cytogenetic findings by fluorescent in situ hybridization (FISH) were available in 299 (81.7%) of 366 versus 440 (80.7%) of 545 patients among two sets of patients. Positive Amp/Gain 1q21 results were observed in 129 (46.9%) versus 215(55.6%) (*p = 0.029*), and positive Del 13q14 results were in 116 (39.6%) versus 215(49.7%) (*p = 0.012*) in patients between the screening and symptom‐driven group. It should be noted that there were 35 (9.6%) and 4 (0.7%) patients with precursor MM who progressed to active MM in the screening and symptom‐driven group, relatively. Other unfavorable factors, including chromosomal abnormalities, Del 17p, t(4;14) and t(14;16), tended to be similar in both groups.

**TABLE 1:** Baseline characteristics of patients diagnosed via two patterns.

Characteristics	Screening‐driven (*n* = 366)	Symptom‐driven (*n* = 545)	*p‐*value
Age, years(range)	66 (35–87)	65 (32–87)	0.093
>65, *n*(%)	188 (51.4)	253 (46.4)	0.156
Male, *n* (%)	239 (65.3)	325 (59.6)	0.084
ISS, *n* (%)			<0.001
I	135 (36.9)	123 (22.6)	
II	114 (31.1)	127 (23.3)	
III	108 (29.5)	285 (52.3)	
Missing	9 (2.5)	10 (1.8)	
IgG phenotype, *n* (%)	222 (61.5)	258 (50.4)	0.001
LDH > 245 U/L, *n* (%)	42 (11.8)	95 (17.8)	0.018
BMPC>60%, *n* (%)	57 (16.1)	186 (35.4)	<0.001
Chromosomal abnormalities, *n* (%)	51 (16.0)	66 (14.1)	0.466
ECOG≧2, *n* (%)	70 (19.1)	264 (47.7)	<0.001
EMD, *n* (%)	58 (15.8)	204 (37.4)	<0.001
CRAB symptom, *n* (%)			
Hypercalcemia	9 (2.5)	43 (8.0)	0.001
Renal Insufficiency	36 (9.9)	102 (18.9)	<0.001
Anemia	113 (31.3)	207 (38.1)	0.034
Bone lesion	281 (77.4)	466 (85.8)	0.001
Cytogenetic abnormalities, *n* (%)			
Del 17p+	25 (8.4)	53 (12.0)	0.113
Del 13q14+	116 (39.6)	215 (49.7)	0.012
Amp/Gain 1q21+	129 (46.9)	215 (55.6)	0.029
t(11;14)	43 (16.0)	64 (16.4)	0.915
t(4;14)	41 (15.1)	74 (18.8)	0.251
t(14;16)	4 (1.5)	13 (3.3)	0.211

*Note*: Student's *t*‐test and Mann–Whitney *U*‐test were used to compare continuous variables in normal and non‐normal distribution status, respectively. The chi‐squared tests were used to compare categorical variables. *p* < 0.05 was considered statistically significant.

Abbreviations: BMPC, bone marrow plasma cells; ECOG, Eastern Cooperative Oncology Group performance status; EMD, extramedullary diseases; ISS, International Staging System; LDH, lactate dehydrogenase.

### 4.3 Treatment information and response

**FIGURE 4:**
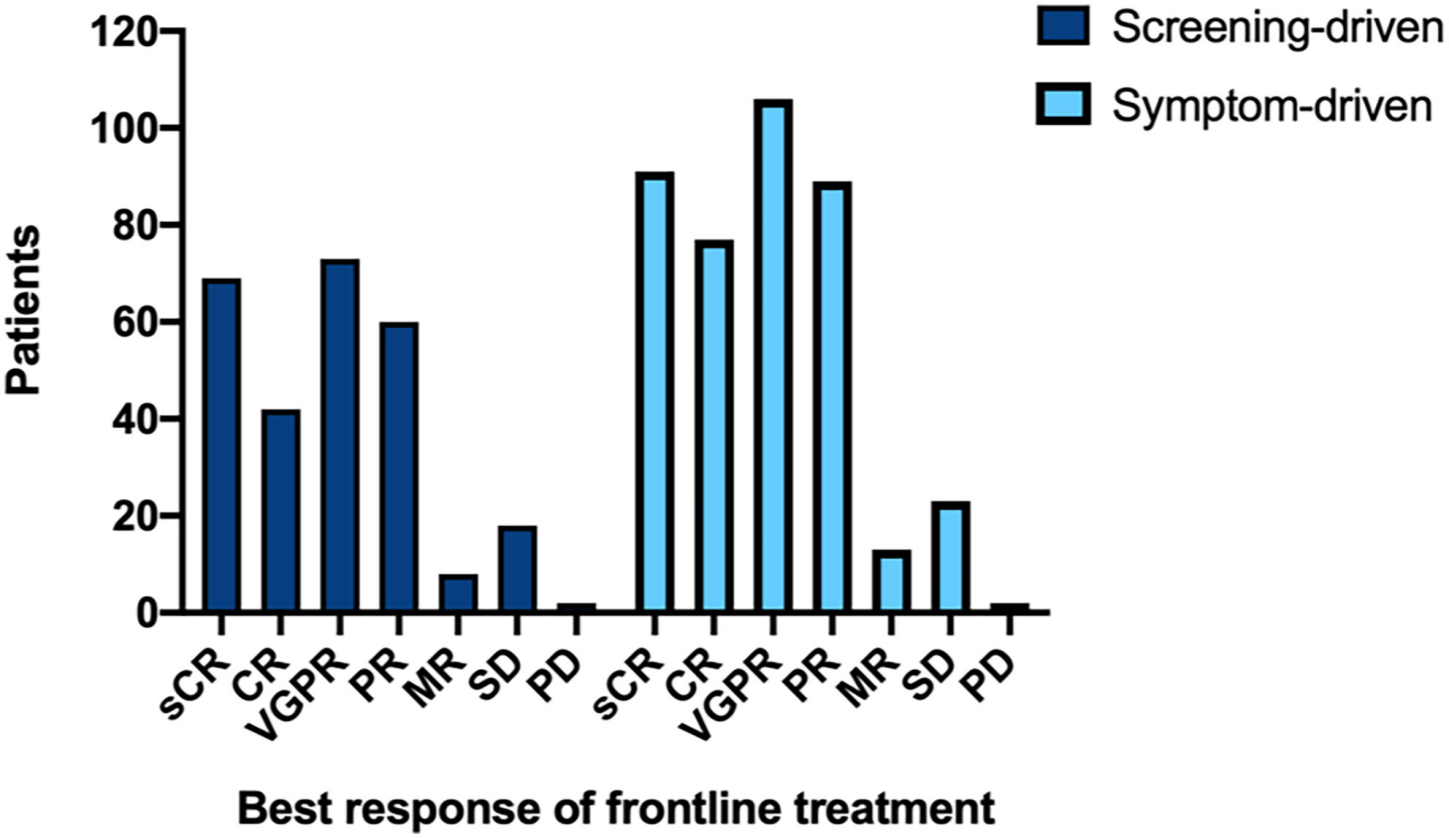
The frontline depth of response in the screening‐driven and symptom‐driven group. Patients who had received at least two cycles of induction therapy were eligible for evaluation of response based on International Myeloma Working Group criteria. There were 272 (74.3%) of 366 patients in the screening‐driven group and 401 (73.6%) of 545 patients in the symptom‐driven group available for response assessment. Best response of frontline treatment here was defined as the assessment after the last induction cycle of first‐line treatment.

Treatment information and response results of two diagnostic groups have been succinctly presented in Table 2. The median number of induction cycles administered was 8 (range, 0–17) and 7 (range, 0–26) for the screening and symptom‐driven group, respectively. A higher proportion of patients (67.8% vs. 60.7%, *p = 0.044*) were administered at least six cycles of induction therapy in the screening group. Of the total cohort, 105 (22.3%) patients under the age of 65 underwent autologous stem cell transplant (ASCT). The symptom‐driven group had a higher rate of ASCT acceptance at 14.9% (*n* = 73), compared to 10.2% (*n* = 33) in the screening group, but this difference was not statistically significant. Patients who received a minimum of two cycles of induction therapy were considered for evaluation of response based on IMWG criteria.^20^ Of the 366 patients in the screening‐driven group and 545 in the symptom‐driven group, 272 (74.3%) and 401 (73.6%) patients, respectively, were available for response assessment. The distribution of response to first‐line therapy is depicted in Figure 4. Among these assessable patients, 184 (67.6%) of 272 in the screening‐driven group and 274 (68.3%) of 401 in the symptom‐driven group achieved a very good partial response (VGPR) or better. Notably, the two groups had no difference in the complete response (CR) rate (40.8% vs. 41.9%, *p* = 0.779). Finally, in the screening and symptom‐driven groups, 137 (37.4%) of 366 patients and 185 (33.9%) of 545 patients, respectively, received maintenance therapy, with no significant difference observed (*p* = 0.280).

**TABLE 2:** Comparison of frontline treatment in two diagnostic groups.

Variables	Screening‐driven *n* = 366	Symptom‐driven *n* = 545	*p‐*value
Number of induction cycle, median(range)	8 (0–17)	7 (0–26)	0.008
≧6 induction cycles, *n* (%)	208 (67.8)	293 (60.7)	0.044
ASCT, *n* (%)	33 (10.2)	73 (14.9)	0.052
>2 drugs regimen, *n* (%)	225 (80.0)	413 (85.0)	<0.001
IMiD exposure, *n* (%)	138 (43.5)	220 (45.2)	0.647
PI exposure, *n* (%)	290 (91.5)	487 (92.2)	0.716
Best depth of response, *n* (%)			
Available	272 (74.3)	401 (73.6)	0.803
≧CR	111 (40.8)	168 (41.9)	0.779
≧VGPR	184 (67.6)	274 (68.3)	0.852
Maintenance therapy	137 (37.4)	185 (33.9)	0.280

*Note*: Student's *t*‐test and Mann–Whitney *U*‐test were used to compare continuous variables in normal and non‐normal distribution status, respectively. The chi‐squared tests were used to compare categorical variables. *p* < 0.05 was considered statistically significant.

Abbreviations: ASCT, autologous stem cell transplant; CR, complete response; IMiD, immunomodulatory drug; PI, proteasome inhibitor; VGPR, very good partial response.

### 4.4 Progression‐free survival and overall survival

**FIGURE 5:**
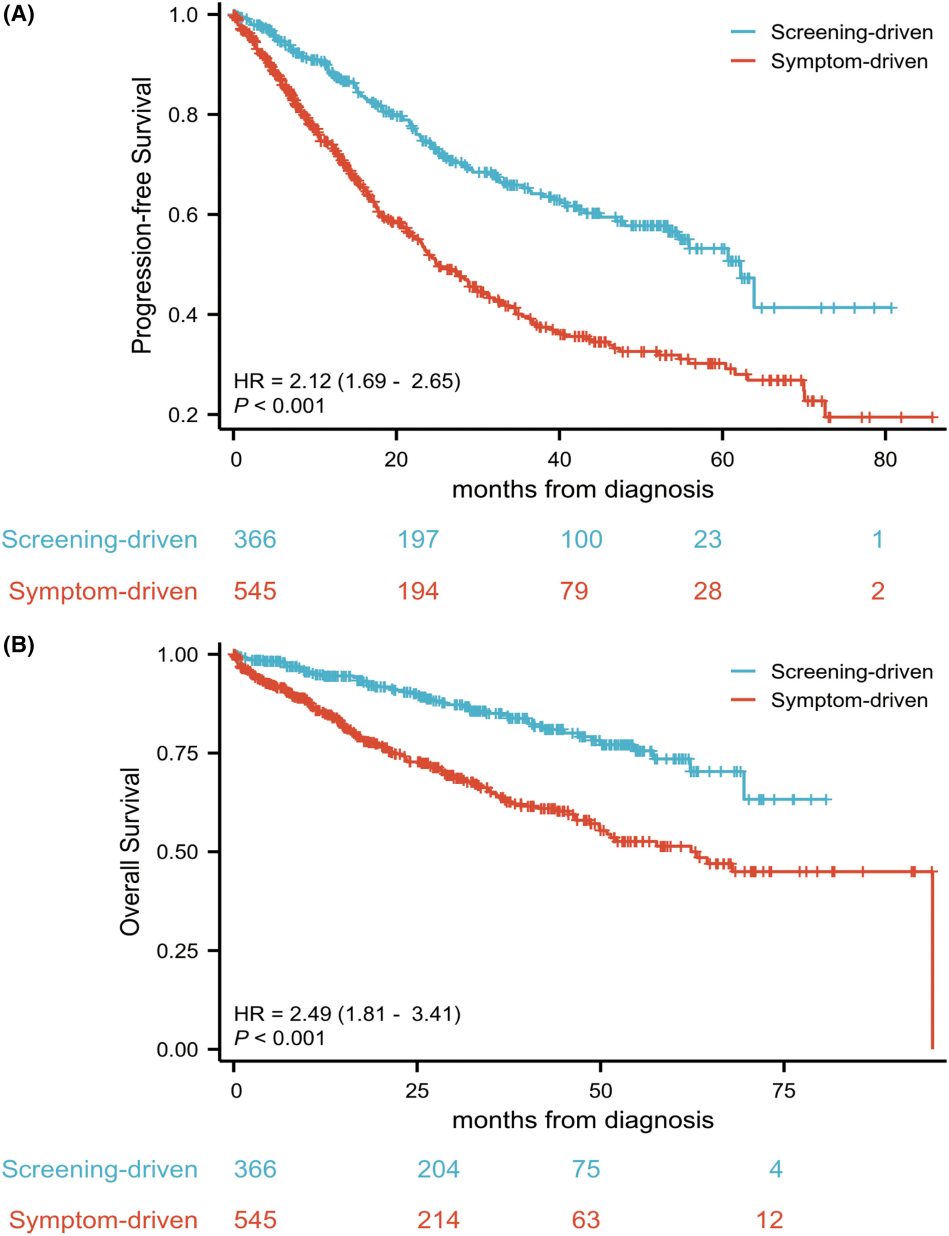
Univariable cox proportional hazards analysis of progression‐free survival (PFS) and overall survival in our cohort. (A, B) Kaplan–Meier analysis of PFS and overall survival (OS) of patients diagnosed through screening (*n* = 366) or symptom (*n* = 545). With respective median follow‐ups of 32.6 and 27.4 months, patients detected via screening had a significantly longer median PFS time than the patients diagnosed via typical symptoms (62.2 vs. 24.9 months respectively). Furthermore, the former group did not achieve the median OS time, which was 62.3 months in the latter group.

With respective median follow‐ups of 32.6 and 27.4 months, there were 108 (29.5%) patients experienced progression and 52 (14.2%) experienced deaths in the screening group at the last follow‐up, compared to 253 (46.4%) progression and 152 (26.1%) deaths in the symptom‐driven group. The median PFS for individuals diagnosed via screening was longer than that for patients diagnosed with myeloma‐related symptoms (62.2 vs. 24.9 months, respectively, *p* < 0.001, HR: 2.12, 95% CIs: 1.69–2.65). The 1‐year/2‐year PFS rates in the former group were 88.5%/74.3%, compared to 74.2%/51.9% in the latter group. The median OS for patients in the screening‐driven group was not reached, while it was 62.3 months in the symptom‐driven group (*p < 0.001*, HR: 2.49, 95% CIs: 1.81–3.41) (Figure 5).

### 4.5 Prognostic factors of the cohort

**FIGURE 6:**
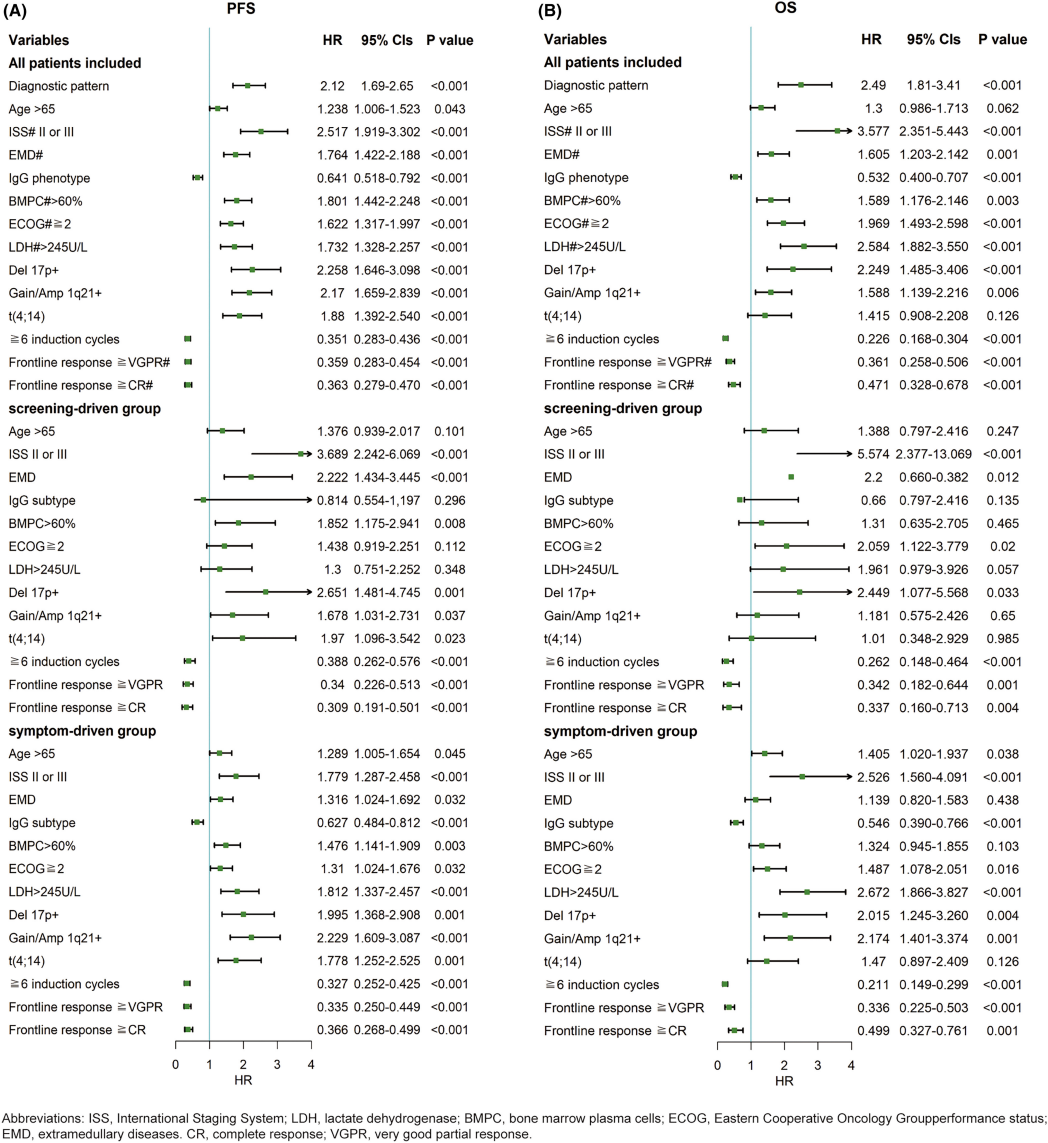
The progression‐free survival (PFS) and overall survival (OS) of patients in two diagnostic subgroups. Univariable cox proportional hazards analyses for PFS (A) and OS (B) among all patients and two diagnostic subgroups were performed. *p* < 0.05 was considered statistically significant.

Cox proportional hazards analyses were performed to assess the association of various factors with both PFS and OS for all patients and two subgroups, as illustrated in Figure 6. Among all patients, advanced ISS stage (II or III), EMD, non‐IgG phenotype, BMPC>60%, ECOG scores of 2 or more, serum LDH level exceeding 245 U/L, and HRCAs (Del 17p and Gain/Amp 1q21) were all associated with poor PFS and OS. In the screening group, ISS stage, EMD, BMPC>60%, and HRCAs (Del 17p, Gain/Amp 1q21, and t(4;14)) were associated with poor PFS, while advanced ISS stage, EMD, ECOG scores of 2 or more, and Del 17p were associated with poor OS. In the symptom‐driven group, all variables included were associated with PFS, while aging over 65, advanced ISS stage, non‐IgG phenotype, ECOG scores of 2 or more, serum LDH > 245 U/L, and HRCAs (Del 17p and Gain/Amp 1q21) were negatively associated with OS. Regarding therapeutic factors, achieving VGPR/CR or better at the first‐line response and receiving at least six cycles of induction therapy were associated with both PFS and OS in the entire cohort and two subgroups.

Further multivariable Cox analyses indicated that diagnostic pattern, ISS stage, EMD, IgG phenotype, serum LDH levels exceeding 245 U/L, Del17p, Gain/Amp 1q21, t(4;14), receiving at least six cycles of induction therapy, and achieving VGPR or better at first‐line response were independent risk factors for PFS among the entire hospital population (Table 3). Meanwhile, diagnostic pattern, ISS stage of II or III, IgG phenotype, serum LDH > 245 U/L, Del17p, Gain/Amp 1q21, receiving at least six cycles of induction therapy, and achieving VGPR or better at first‐line response were independently predictive of OS (Table 4). For the screening group, prognostic factors of PFS were ISS stage, EMD, t(4;14), and achieving VGPR/CR or better at first‐line response. On the other hand, for the symptom‐driven group, IgG phenotype, serum LDH > 245 U/L, Del17p, t(4;14), receiving at least six cycles of induction therapy, and achieving CR or better at first‐line response were indicative of PFS (Table 3). Only the ISS stage was independently associated with OS in the screening group due to the limited number of events. ISS stage, IgG phenotype, serum LDH > 245 U/L, Del17p, Gain/Amp 1q21, and receiving at least six cycles of induction therapy showed an independent impact on OS of patients in the symptom‐driven group (Table 4).

**TABLE 3:** Multivariable cox proportional hazards analysis of progression‐free survival (PFS) in our cohort.

Variables	All patients included			Screening‐driven group			Symptom‐driven group		
	Hazard ratio	95% CIs	*p‐*value	Hazard ratio	95% CIs	*p‐*value	Hazard ratio	95% CIs	*p‐*value
Diagnostic pattern	1.906	1.293–2.81	0.001	‐			‐		
Age > 65	1.074	0.781–1.476	0.662	‐			1.042	0.713–1.522	0.831
ISS II or III	1.938	1.25–3.005	0.003	2.275	1.111–4.659	0.025	1.579	0.908–2.748	0.106
EMD	1.507	1.048–2.167	0.027	4.69	2.055–10.705	<0.001	1.158	0.774–1.732	0.475
IgG phenotype	0.6	0.431–0.835	0.002	‐			0.52	0.348–0.776	0.001
BMPC>60%	1.155	0.81–1.647	0.425	0.86	0.382–1.939	0.717	1.178	0.783–1.773	0.431
ECOG≧2	0.933	0.661–1.317	0.693	‐			0.893	0.605–1.317	0.567
LDH > 245 U/L	1.68	1.072–2.632	0.023	‐			2.148	1.276–3.618	0.004
Del 17p+	2.578	1.575–4.221	<0.001	2.303	0.83–6.389	0.109	2.423	1.38–4.256	0.002
Gain/Amp 1q21+	1.55	1.092–2.199	0.014	1.554	0.817–2.956	0.179	1.525	0.98–2.372	0.061
t(4;14)	2.11	1.48–3.008	<0.001	2.367	1.108–5.057	0.026	2.244	1.454–3.461	<0.001
≧6 induction cycles	0.516	0.357–0.745	<0.001	0.562	0.282–1.121	0.102	0.466	0.301–0.723	0.001
Frontline response ≧VGPR	0.657	0.443–0.975	0.037	0.445	0.21–0.945	0.035	0.759	0.474–1.217	0.252
Frontline response ≧CR	0.446	0.292–0.682	<0.001	0.364	0.149–0.893	0.027	0.457	0.28–0.745	0.002

Abbreviations: BMPC, bone marrow plasma cells; CR, complete response; ECOG, Eastern Cooperative Oncology Group performance status; EMD, extramedullary diseases. ISS, International Staging System; LDH, lactate dehydrogenase; VGPR, very good partial response.

**TABLE 4:** Multivariable cox proportional hazards analysis of overall survival (OS) in our cohort.

Variables	All patients included			Screening‐driven group			Symptom‐driven group		
	Hazard ratio	95% CIs	*p‐*value	Hazard ratio	95% CIs	*p‐*value	Hazard ratio	95% CIs	*p‐*value
Diagnostic pattern	2.151	1.251–3.698	0.006	‐			‐		
Age > 65	‐			‐			1.152	0.696–1.905	0.582
ISS II or III	4.08	1.947–8.549	<0.001	3.509	1.276–9.646	0.015	4.028	1.558–10.412	0.004
EMD	1.063	0.638–1.771	0.815	2.191	0.839–5.721	0.109	‐		
IgG phenotype	0.511	0.316–0.826	0.006	‐			0.468	0.266–0.824	0.009
BMPC>60%	0.763	0.464–1.253	0.285	‐			‐		
ECOG≧2	1.42	0.893–2.258	0.138	1.916	0.809–4.535	0.139	1.183	0.715–1.957	0.512
LDH > 245 U/L	1.83	1.039–3.225	0.036	‐			1.933	1.053–3.549	0.034
Del 17p+	1.882	1.006–3.522	0.048	1.561	0.581–4.19	0.377	2.826	1.394–5.728	0.004
Gain/Amp 1q21+	1.736	1.068–2.824	0.026	‐			2.272	1.247–4.14	0.007
t(4;14)	‐			‐			‐		
≧6 induction cycles	0.345	0.207–0.574	<0.001	0.433	0.178–1.051	0.064	0.328	0.186–0.58	<0.001
Frontline response ≧VGPR	0.495	0.271–0.903	0.022	0.433	0.151–1.243	0.12	0.4	0.249–1.003	0.051
Frontline response ≧CR	1.039	0.569–1.897	0.901	0.77	0.29–2.047	0.6	1.405	0.692–2.852	0.346

Abbreviations: BMPC, bone marrow plasma cells; CR, complete response; ECOG, Eastern Cooperative Oncology Group performance status; EMD, extramedullary diseases. ISS, International Staging System; LDH, lactate dehydrogenase; VGPR, very good partial response.

## 5 DISCUSSION

MM poses a formidable challenge as a hematological malignancy characterized by a gradual progression. Previous research has shown that timely detection of myeloma leads to better clinical outcomes and minimizes the chances of disease recurrence.^12^, ^22^, ^23^ Conducting a population‐based screening for MM holds the potential to pinpoint the onset of the disease prior to the onset of complications.^14^ Therefore, commitments were made to perform MGUS screening in various regions.^24^ However, evidence on the patient outcomes of screening programs is still limited, and prospective trials such as iStopMM are still under investigation.^10^ Our study collected 7‐year real‐world data in our institute to interpret the association between hospital‐based screening and survival benefits of MM patients.

The M‐protein screening system was initially carried out in 2014 at Zhongshan Hospital.^17^ With SPEP integrated into standard biochemistry tests (liver function tests), hundreds of thousands of screening tests were performed annually. The sizeable number of samples provided compelling evidence as well as challenges. Digital techniques used in this program helped us with advanced warnings and timely interventions for suspected patients. For the patient having a positive SPEP result, the healthcare provider could receive a prompt in the result report, thereby suggesting the patient to have additional examinations at the next visit to our hospital. Furthermore, the EDC system enabled us to collect streamlined follow‐up information, thus constructing two study cohorts: the screening‐driven and the symptom‐driven group. In our study, it should be noted that the incidence rate of MM in the hospital screening population was much higher than reported before.^17^ Compared to 1.03/100,000 in China,^5^ the incidence of NDMM among patients participating in the M‐protein screening program was 31.9/100,000. Considering the study was performed in the hospital population, the actual incidence could have been over‐estimated due to selection bias. But the massive difference in incidence (1.03/100,000 vs. 31.9/100,000) could imply the underestimation of actual NDMM prevalence in China due to miss diagnosis or delayed diagnosis. Thereby, the M‐protein screening may improve the detection rate of MM.

The baseline characteristics of NDMM patients in our cohort were similar to those reported in previous real‐world studies.^25^, ^26^ One noteworthy finding of our study was that patients who underwent screening were diagnosed at the early stage, with fewer CRAB symptoms, earlier ISS stages and fewer CAs. After extending the follow‐up period and analyzing nearly three times as many patients,^17^ we also observed that patients in the screening‐driven group had improved PFS and OS than those in the diagnosis‐delayed symptom‐driven group. In order to identify important variables affecting patient outcomes, cox proportional hazards analyses for PFS and OS were conducted. Among all patients, the diagnostic pattern was significantly related to prolonged PFS and OS after being adjusted by well‐acknowledged myeloma prognostic factors (Tables 3 and 4). It convinced us that early detection of MM by screening in the hospital population resulted in superior outcomes for patients. For details of univariable and multivariable cox hazards models, the ISS stage was an independent prognostic factor of OS in both groups. At the same time, LDH, M‐protein phenotype, Del17p and Gain/Amp 1q21 were predictive of OS in the symptom‐driven group. Meanwhile, t(4;14) were independently related to worse PFS in both groups and IgG phenotype, LDH and Del17p were related to PFS in the symptom‐driven group. It provided evidence that the screening diagnostic pattern may overcome the negative effect of some unfavorable prognostic factors for MM.

In addition, there were 35 patients progressing to active MM in the screening group, which was four in the symptom‐driven group, and most of patients with MGUS or SMM detected via screening before would be followed up regularly. As reported,^13^ MM patients with a history of monoclonal gammopathy had a lower mortality rate than those without, probably as a result of better follow‐up and early detection of disease progression. These reasons may also partially contribute to better survival outcomes for our MM patients in the screening‐driven group.

Regarding therapy, a prior study^27^ showed that even achievement of CR was not associated with prolonged PFS in NDMM patients. However, our study revealed that attaining a deep response during frontline therapy (≧VGPR/CR) was an independent prognostic factor for PFS in both groups (Tables 3 and 4). Furthermore, in the symptom‐driven group, PFS and OS were enhanced by extending the duration of induction cycles. The findings suggest that adherence to standard induction therapy and achieving a more profound response is clinically significant for patients with advanced disease stages. Further research is needed to explore additional prognostic factors that may impact the outcomes of NDMM patients. As for ASCT, 105 (22.3%) patients under 65 received ASCT in the whole cohort. It showed a lower rate of patients receiving ASCT than other studies in developed countries, such as Australia/New Zealand.^28^ It is also reported at 24.6% in a Japanese study^26^ and 26.2% in another Chinese cohort of ASCT rate.^29^ As a previous study by our team explained,^30^ Chinese patients hesitated to receive ASCT mainly because of their preference, financial concerns, and fear of side effects, which promoted them to choose non‐transplantation regimens.

To our knowledge, it was the first real‐world report to validate the efficacy of screening in the hospital population. With the seven‐year follow‐up, we summarized the characteristics of NDMM patients from different diagnostic patterns and reported the outcome information in China. However, there may have some limitations. (1) First, this was a retrospective study conducted at a single center, which may limit the generalizability of our findings to other settings. (2) Additionally, selection bias may affect our results as the symptom‐driven group consisted of patients who presented with disease symptoms, indicating differences in disease severity and medical accessibility between the two groups. (3) Our screening program recommended a serum free light chains test after a positive SPEP result. Using only SPEP as the initial step for detecting M proteins can result in a lower detection rate for patients with light chain only disease. However, as a screening test, we can only use inexpensive tests to screen as many people as possible, rather than using the most accurate tests to screen the entire population.

## 6 CONCLUSION

In this study, we provided a summary of the 7‐year screening initiatives for newly NDMM in the hospital population, and presented the follow‐up results for patients who were diagnosed either through screening or based on symptomatic patterns. Our findings validate that screening for M‐protein facilitates early detection and enhances the prognosis for patients with MM.

## AUTHOR CONTRIBUTIONS

**Wenjing Wang:** Data curation (equal); formal analysis (lead); resources (equal); writing – original draft (lead). **Jing Li:** Investigation (equal); project administration (lead); software (equal); writing – review and editing (equal). **Yang Yang:** Data curation (equal); project administration (equal); resources (equal). **Feifei Chen:** Data curation (equal); investigation (equal). **Tianhong Xu:** Data curation (equal). **Pu Wang:** Data curation (equal). **Yawen Wang:** Data curation (equal). **Aziguli Maihemaiti:** Data curation (equal). **Liang Ren:** Data curation (equal). **Tianwei Lan:** Data curation (equal). **Panpan Li:** Data curation (equal). **Chi Zhou:** Data curation (equal). **Peng Liu:** Conceptualization (lead); funding acquisition (lead); methodology (lead); resources (lead).

## FUNDING INFORMATION

This work has been supported by the Natural Science Foundation of Shanghai (22ZR1411400).

## CONFLICT OF INTEREST STATEMENT

The authors declare that the research was conducted in the absence of any commercial or financial relationships that could be construed as a potential conflict of interest.

## ETHICS STATEMENTS

No animal studies are presented in this manuscript. The studies involving human participants were reviewed and approved by the clinical ethics committee of Zhongshan Hospital Fudan University. The patients/participants provided their written informed consent to participate in this study. No potentially identifiable human images or data is presented in this study.

## Data Availability

The raw data supporting the conclusions of this article are available from the corresponding author upon reasonable request.
